# Impact of Inappropriate Empiric Antimicrobial Therapy on Mortality of Septic Patients with Bacteremia: A Retrospective Study

**DOI:** 10.1155/2012/765205

**Published:** 2012-08-02

**Authors:** Saoraya Lueangarun, Amorn Leelarasamee

**Affiliations:** ^1^Department of Medicine, Chulabhorn Hospital, Lak Si, Bangkok 10210, Thailand; ^2^Department of Medicine, Faculty of Medicine Siriraj Hospital, Mahidol University, Bangkok 10700, Thailand

## Abstract

*Background*. Inappropriate empiric antimicrobials could be a major cause of unfavorable mortality rates in co-morbid patients. This study aimed to assess the prevalence and impact of first-dose and 24-hour inappropriate antimicrobials on mortality rates of bacteremic septic patients. *Methods*. A retrospective cohort study was employed. Case record forms of patients diagnosed as sepsis, severe sepsis, or septic shock with positive hemoculture during 2009 were retrieved from the medical wards, Siriraj Hospital. Demographic data, antimicrobial use, types of bacteria isolated from blood and susceptibilities, patients' comorbidities, 28-day and overall mortality rates were collected and analyzed. *Results*. There were 229 cases, mean age (SD) of 63.5 (17.2) years and mean (SD) APACHE II score of 24.7 (6.8). The prevalence of first-dose and 24-hour inappropriate antimicrobials was 29.7% and 25.3%, respectively. The 28-day and overall mortality rates between first-dose inappropriate and appropriate antimicrobial were 67.6% versus 60.2% (*P* = 0.301) and 75.0% versus 68.3% (*P* = 0.345), consequently. Patients with septic shock and inappropriate first-dose antimicrobials significantly had higher 28-day mortality rate (61.6% versus 41.9%; *P* = 0.017). *Conclusion*. Higher mortality rates in bacteremic septic patients were substantially associated with inappropriate first-dose antimicrobials and 3-hour delayed antimicrobial administration after sepsis diagnosis.

## 1. Background

Sepsis is one of the most serious conditions related to high mortality in approximately 0.1–5 per 100 cases admitted to the hospital, and it also accounts for 5–15 percent of cases with overall infections. In 2007, there were 201 (5.8%) cases diagnosed as sepsis from 3,451 patients admitted to the medical wards in the Siriraj Hospital, of which 38.8% developed septic shock. Inappropriate antimicrobial therapy administration during the first 24 hours was observed in 34.2%. The mortality rate of patients with sepsis and septic shock was as high as 34.3% and 52.6%, respectively [1].

Two important factors on antimicrobial therapy pertaining to adverse events and death in septic patients were the initiation of inappropriate antimicrobial therapy [2] and the delay of appropriate antimicrobial therapy [3]. Inappropriate empiric antimicrobial therapy was attributed to 46.5% of cases, with 35% overall mortality [3]. The elapsed time to appropriate antimicrobial therapy was crucial for the mortality in patients with severe sepsis and septic shock [4]. The Surviving Sepsis Campaign's 2008 “International guidelines for the management of severe sepsis and septic shock” recommended that appropriate antimicrobial therapy should be administered within 1 hour of severe sepsis or septic shock recognition [3, 5].

Hence, we aimed to assess the prevalence and impact of inappropriate first-dose antimicrobial therapy and delayed antimicrobial administration on the mortality of patients with sepsis. Also, the risk factors associated with inappropriate antimicrobial therapy and high mortality rate in these patients were determined.

## 2. Materials and Methods

### 2.1. Study Design and Population

A retrospective cohort study was conducted during January–December 2009 at the medical wards of the Siriraj Hospital. We enrolled the patients by looking at their positive blood cultures first and then searched for those who did have clinical sepsis by looking at the patients' charts. Patients diagnosed as sepsis, severe sepsis, or septic shock, with positive hemoculture on the day of diagnosis, were included. We excluded the patients with second episode of sepsis or more likely with bacteremia in the same admission, polymicrobial infection, and organisms other than bacteria (e.g., fungus). All cases with positive hemoculture result were determined to meet the specific criteria for sepsis, severe sepsis, and septic shock according to the American College of Chest Physicians/Society of Critical Care Medicine (ACCP/SCCM) consensus conference definition [6]. Data were collected from inpatient-recording and drug-prescribing charts by the first author, using standardized case record forms. All data were collected from inpatient-recording charts and drug-prescribing chart by first researcher using a standardized case record forms. Data collection included patients' demographics, co-existing conditions, diagnosis of sepsis (severe sepsis or septic shock), Acute Physiology and Chronic Health Evaluation (APACHE) II score [7], site of infection, causative bacteria, antimicrobial usage, timing of blood culture collection, actual time of initial parenteral antimicrobial administration, clinical course, and treatment outcome. Diagnosis timing of sepsis was defined as the time for positive hemoculture performance. Timing of first-dose and 24-hour antimicrobial therapy was retrieved from drug-prescribing charts. The 24-hour antimicrobial adjustment was at the physicians' own discretions. Questionable cases or data elements were reviewed and adjusted by the principal investigator.

### 2.2. Definitions

The definitions of clinical infection, systemic inflammatory response syndrome (SIRS), sepsis, severe sepsis, and septic shock were adapted from previous recommendations and studies [6, 8–11].

Bacteremia was defined as the presence of viable bacteria in blood, detected by positive hemoculture. Severity of diseases was classified as sepsis, severe sepsis, and septic shock according to previous definitions [6] and APACHE II scores, determined by the highest abnormal data within 24 hours of sepsis onset [7].

Community-acquired and nosocomial infections were defined as previous studies [8]. Healthcare-associated infection was defined as an infection in a patient being hospitalized for more than 2 days in the preceding 90 days or a resident in a nursing home or extended care facility, with home infusion therapy, chronic dialysis treatment within 30 days, or home wound care [12].

Timing of the diagnosis of sepsis was defined as the time when positive blood culture was performed, and then signs and symptoms of sepsis of these patients must be present when their clinical status were retrieved from the patient record chart at that time point. Hence, the patients with subsequent positive blood cultures and presence of clinical criteria of sepsis, severe sepsis, or septic shock were enrolled in our study. Timing of first and 24-hour dose antimicrobial therapy, defined as duration from diagnosis of sepsis to antimicrobials administration, retrieved from drug-prescribing charts.

Drug-resistant gram-positive bacteria included methicillin-resistant *Staphylococcus aureus* (MRSA) and *Enterococcus* spp.. Drug-resistant gram-negative bacteria were extended-spectrum *β*-lactamase (ESBL)-producing Enterobacteriaclae, *Acinetobacter* spp., and *Pseudomonas aeruginosa.*

Appropriate antimicrobial therapy was defined as the isolated bacteria being susceptible to at least one of the antimicrobials empirically administered as the first dose or 24 hours later. In the absence of specific sensitivity testing, the followings were considered as appropriate therapy: (a) group A, B, and G *Streptococcus* treated with all beta-lactams; (b) all gram-positive bacteria except enterococci treated with vancomycin. Meanwhile, in the absence of specific sensitivity testing, the enterococci treated with all cephalosporin and trimethoprim/sulfamethoxazole were considered as inappropriate therapy [2, 11, 13].

### 2.3. Objective

The primary objectives were to determine the prevalence of first-dose and 24-hour inappropriate empiric antimicrobial therapy in septic patients with bacteremia and its impact on 28-day and overall mortality rates. The secondary objectives were to define the appropriate timing of antimicrobial administration and factors associated with inappropriate antimicrobial therapy and high mortality rate in bacteremic patients with sepsis.

### 2.4. Statistical Analysis

Descriptive statistics were implemented to summarize the patients' characteristics as mean (SD) or proportions. Chi-square or Fisher's exact test for the categorical data and Student's *t*-test or Mann-Whitney *U* test for the continuous data were employed to compare data between different groups. Multiple logistic regression analysis of independent factors associated with 28-day and overall mortality rates and factors related to inappropriate empirical antimicrobial treatment of bacteremia was performed. A *P*-value of 0.05 or less was considered statistically significant. SPSS version 18.0 software was used for data analysis.

## 3. Results

### 3.1. Demographic Data

A total of 229 cases with bacteremia were eligible for the specific study criteria. The mean (SD) age of septic patients was 63.5 (17.2) years, with 49.8% male patients. The mean (SD) APACHE II score was 24.7 (6.8). Comorbidities were frequently presented in 96.9% of the patients. The common comorbidities were diabetes mellitus (31.0%), immunosuppressive therapy (29.3%), reduced mobility (29.7%), liver failure (21.8%), congestive heart failure (21.8%), chronic kidney disease (18.3%), and hematologic malignancy (18.2%) (Table 1).

### 3.2. Pattern of Infections

Types of infection were community-acquired (27.5%), healthcare-associated (37.1%), and nosocomial infections (35.4%). Septic shock and severe sepsis were present in 61.1% and 25.3% of the enrolled patients. The common sites of infection were respiratory tract (32.8%), gastrointestinal tract (23.6%), and genitourinary tract (20.5%) (Table 2).

### 3.3. Causative Pathogens

The most commonly identified bloodstream pathogens were Gram-negative bacteria (72.5%) as follows:* Escherichia coli *(28.2%),* Klebsiella pneumoniae *(12.7%), and *Acinetobacter* spp. (12.2%). gram-positive bacteria were found in 27.5% of cases, including methicillin-susceptible *S. aureus *(MSSA) (8.7%), methicillin-resistant *S. aureus *(MRSA) (4.8%), and *Streptococcus* group D (3.5%). Drug-resistant gram-positive bacteria, gram-negative bacteria, ESBL-producing organism, and ESKAPE organism [14] were observed in 7.4%, 32.3%, 15.7%, and 73.8% of the isolates, respectively (Table 3).

### 3.4. Type of Empiric Antimicrobial Treatment

About 63.3% of septic patients received single antimicrobial therapy. Antimicrobials frequently administered were cephalosporin (57.6%), carbapenem (23.1%), beta-lactam/beta-lactamase inhibitor (12.2%), vancomycin (11.4%), aminoglycosides (7.4%), fluoroquinolones (8.3%), and colistin (4.8%). Only colistin significantly associated with appropriate first-dose antimicrobial therapy (6.8% versus 0%, *P* < 0.05) (Table 4).

### 3.5. Inappropriateness of Empiric Initial Antimicrobial Therapy

**Figure 1:**
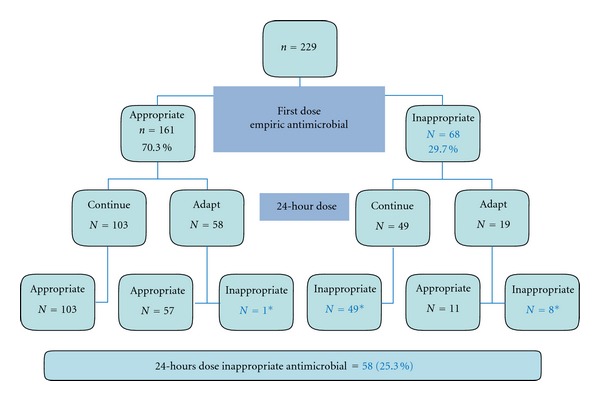
Number and percentage of inappropriate empiric antimicrobial therapy at first dose and subsequent adaptation at 24 hours (Cont; Continuation of Empiric Antimicrobial Therapy, Adapt; Adaptation of Empiric Antimicrobial Therapy).

Among 229 patients, 143 and 161 cases died within 28 days and during the hospitalization, subsequently. In general, the 28-day and overall mortality rates were 62.4% and 70.3%, respectively. Inappropriate first-dose and 24-hour empiric antimicrobial therapies were consequently noted in 68 cases (29.7%) and 58 cases (25.3%) of the patients. At 24 hours, 58 cases in the appropriate group and 19 cases in the inappropriate group had their empiric antimicrobials adapted. At 24 hours of diagnosis, one case in the appropriate group and 8 cases in the inappropriate group were still treated with inappropriate antimicrobials (Figure 1).

When compared to appropriate antimicrobial therapy, inappropriate first-dose antimicrobial therapy tended to have higher 28-day and overall mortality rates (67.6% versus 60.2% (*P* = 0.301) and 75.0% versus 68.3% (*P* = 0.345), resp.). Additionally, inappropriate 24-hour empirical antimicrobials were also likely to have higher 28-day and overall mortality rates (65.5 versus 61.4% (*P* = 0.64) and 74.1 versus 69.0% (*P* = 0.51), resp.). Univariate analysis of factors associated with 28-day and overall hospital mortalities in all sepsis patients was shown in Table 5.

There were significant variations in the appropriateness of initial antimicrobial therapy between the groups of clinical infections and isolated organisms. Nosocomial infection (37.0%) appeared to have significantly higher inappropriate antimicrobials than community-acquired (14.3%) and healthcare-associated infection (22.3%, *P* = 0.004). Drug-resistant gram-positive bacteria (MRSA and Enterococci) and drug-resistant gram-negative bacteria (*Acinetobacter spp*.,* Pseudomonas aeruginosa*,and ESBL-producing organisms) were significantly related to inappropriate first-dose empirical antimicrobials (64.7% versus 35.3%, *P* = 0.002 and 62.2% versus 37.8%, *P* = 0.001, resp.). Subgroup analyses of drug-resistant organisms also showed a significant relation with the inappropriateness of first-dose antimicrobial therapies, such as *Acinetobacter* spp. (60.7% versus 39.3%, *P* < 0.001), ESKAPE organism (*Enterococcus faecium*, *Staphylococcus aureus*,* Klebsiella pneumoniae*,* Acinetobacter baumannii*,* Pseudomonas aeruginosa*, *Enterobacter* spp. (65.1% versus 34.9%, *P* < 0.001), and extended-spectrum *β*-lactamase (ESBL)-producing organisms (77.8% versus 22.2%, *P* < 0.001). Moreover, both drug-resistant gram-positive and gram-negative bacteria significantly had higher inappropriate antimicrobial rates (64.7% versus 35.3%; *P* = 0.002 and 62.2% versus 37.8%; *P* = 0.001, resp.).

Patients with genitourinary tract and skin and soft tissue infections appeared to have lower rate of inappropriate antimicrobial therapy than those with infections at other sites (57.4% versus 42.6%; *P* = 0.048 and 94.4% versus 5.6%; *P* = 0.016, resp.).

From multivariate analysis, factors significantly associated with the inappropriateness of first-dose empirical antimicrobials included drug-resistant Gram-positive organism (OR 6.03, 95% CI 2.35–15.43), drug-resistant Gram-negative organism (OR 10.76, 95% CI 3.98–29.09), leukopenia (OR 2.45, 95% CI 1.18–5.08), neutropenia (OR 2.94, 95% CI 1.16–7.14), and platelet < 100,000 per mm^3^ (OR 2.42, 95% CI 1.20–4.88) (Table 6).

### 3.6. Impact of Inappropriate Initial Empiric Antimicrobial Therapy Administration

At hospital discharge, patients with inappropriate initial empiric antimicrobial therapy had a trend towards higher mortality rate than those with appropriate empiric antimicrobial therapy (75.0% versus 68.3%; *P* = 0.345). In addition, the first-dose and 24-hour inappropriate antimicrobial therapy resulted in shorter median survival duration than the appropriate antimicrobial therapy administration (214.7 hours versus 301.1 hours, *P* = 0.86 and 180.2 hours versus 301.1 hours, *P* = 0.85, resp.).

Multivariate analysis of factors potentially associated with fatal outcome revealed that the inappropriateness of the first-dose antimicrobial therapy administration remained the most strongly correlated with high mortality (OR 2.52; 95% CI 1.01–6.32; *P* = 0.049) among all the factors assessed. Other factors related to overall mortality included the timing from diagnosis until the first-dose antimicrobial therapy, septic shock status, presence of congestive heart failure, age >65 years, APACHE II score >25, platelet count < 100,000 per mm^3^, and serum albumin < 3.2 g/dL (Table 7).

### 3.7. Impact of Timing from Diagnosis Until First-Dose Antimicrobial Therapy on Overall Mortality

Antimicrobials were initiated within 1 hour and 6 hours after onset of sepsis in 20.1% and 64.2% of cases, respectively. Nevertheless, 9.6% of the patients received antimicrobial therapy after 24 hours of sepsis onset.

The mean (SD) length of time from onset of diagnosis to the first-dose antimicrobial therapy was 9.57 (25.33) hours in all cases and 7.00 (16.61) hours in those with appropriate antimicrobial therapy. The patients with appropriate antimicrobial therapy were significantly administered to receive antimicrobials earlier than those treated with inappropriate antimicrobials (7.00 (16.60) versus 15.66 (38.37) hours, *P* = 0.018).

All patients and those with appropriate antimicrobial therapy in the 28-day and overall hospital mortality group were likely to have delayed first-dose antimicrobials. In all patients, the mean (SD) duration from onset of sepsis to appropriate antimicrobial therapy of the 28-day and overall mortality group was longer than the 28-day and overall survival group (7.09 (18.61) versus 6.85 (13.15) hours, *P* = 0.931 and 7.14 (18.48) versus 6.68 (11.73) hours, *P* = 0.931, resp.). In patients with appropriate antimicrobials, the mean (SD) duration from onset of sepsis to appropriate antimicrobial therapy in the 28-day and overall mortality group was also more prolonged than the 28-day and overall survival group (9.89 (27.69) versus 9.03 (20.99) hours, *P* = 0.806 and 10.27 (27.30) versus 7.91 (19.99) hours, *P* = 0.522, resp.).

There was significant relationship between the delayed timing from diagnosis to the administration of first-dose antimicrobials and the overall mortality. The mortality rates in patients with antimicrobials less than 1 hour, from 1 hour to 6 hours, and more than 6 hours after the diagnosis of sepsis were 63.0%, 65.3%, and 80.5%, respectively (*P* = 0.04). Also, higher overall mortality was demonstrated after the delayed antimicrobial initiation over 1 hour (72.1 versus 48.7%, *P* = 0.30). However, significantly higher overall mortality was observed in those with antimicrobial therapy more than 3 hours after the diagnosis (OR 1.92, 95% CI 1.08–3.42).

## 4. Discussion

Our data strongly supported the notion that the inappropriate antimicrobials and the delayed administration of appropriate antimicrobial therapy could substantially increase the mortality rate in septic patients with bacteremia. We reported herein that the inappropriate empirical antimicrobial therapy was frequent among bacteremic patients with high overall mortality and a tendency towards higher mortality rate than the appropriate antimicrobial therapy. This similar high percentages of inappropriate antimicrobial therapy, ranging from 20% to 46.5%, were also described by several previous reports, which resulted in high mortality rate from 35% to 70% [1, 2, 17–27].

However, the overall mortality rate in our study was higher than the one conducted in the same hospital during 2007 [1]. The difference in mortality rates between these two studies could be explained by distinctive population groups, higher percentages of healthcare-related and nosocomial infections, and more severity of infections in the latter study. The study conducted in 2007 [1] enrolled patients with clinical entities of sepsis, severe sepsis, or septic shock regardless of whether they were bacteremic or not at the entry point. Meanwhile, the study in 2009 recruited patients with bacteremia and clinical sepsis, severe sepsis, or septic shock. The population study groups in 2007 consisted of sepsis patients with bacteremia (40.8%), community-acquired infection (61.2%), and septic shock (38.8%). On the contrary, our study populations were bacteremic patients with healthcare-related infection (37.1%), nosocomial infection (35.4%), and septic shock (61.1%). Hence, there was more severity of infections in our study population groups that led to higher mortality rate.

Other factors pertaining to higher mortality rate included microbiological factors such as the presence of frequent healthcare-associated and nosocomial infections, and drug resistant organisms. Despite multiple risk factors for high mortality in sepsis patients, the inappropriate first-dose empirical antimicrobial therapy still significantly increased 6.7% of mortality rate. The results in our study corresponded to those of Kumar et al., demonstrating higher mortality among septic shock patients with inappropriate empirical antimicrobials (89.7% versus 48.0%) [2]. Nevertheless, the impact of inappropriate empirical antimicrobials in patients with less Comorbidities and severity of sepsis remained to be elucidated. There were also significant variations in the inappropriateness of initial antimicrobial therapy among infection sites, of which genitourinary and skin and soft tissue infections seemed to have appreciably better empirical antimicrobial therapies.

Inappropriate empiric antimicrobial therapy was notably higher in the gram-negative bacteria group than the gram-positive bacteria group, resulting in the increasing of mortality in gram-negative bacteremia, especially drug-resistant organisms as reported by Kang et al. that inappropriate antimicrobial therapy in gram-negative septicemia had higher 30-day mortality rate (38 versus 27%, *P* < 0.05) [28].

One of the major causes of inappropriate initial antimicrobial therapy was the underrecognition of the infections with antimicrobial-resistant organisms [2, 21, 24, 29–31], of which drug-resistant gram-negative bacteria significantly associated with inappropriateness empirical antimicrobials. Especially, ESBL-producing *Escherichia coli* (*E. coli*) and *Klebsiella pneumonia* (*K. pneumoniae*) rapidly spread worldwide and became a serious healthcare problem [32]. The Thai prevalence of ESBL-producing *E. coli* and ESBL-producing *K*. *pneumoniae* in healthcare-associated infections was 13.2% and 12.7%, respectively [33]. From our study, ESBL-producing Enterobacteriacae accounted for 15.7% of bacteremia, which considerably associated with both first-dose and 24-hour inappropriate empirical antimicrobials. *E. coli* was the most commonly conducting to bacteremia in our study, of which 42.4% were ESBL-positive organisms that led to more inappropriate antimicrobials than ESBL-negative organisms. Consequently, the patients' risk factors for harboring ESBL-producing resistant organisms, including length of hospital or ICU stay, central venous or arterial catheters [34], emergency intra-abdominal surgery, gastrostomy or jejunostomy tube, colonization of ESBL-producing organisms in the gastrointestinal tract [34, 35], prior administration of any antibiotics [36], prior long-term care facility stay, severity of diseases, urinary catheter [37], ventilatory assistance, and hemodialysis, should be considered in order that treatment with carbapenem antimicrobial in producing the better outcomes of survival and bacteriologic clearance [38, 39] would be more implemented [40].

*Acinetobacter* spp., multidrug-resistant strains as common causes of nosocomial infection and important antimicrobial-resistant organism [32, 41, 42], was 12.2% frequently found and significantly associated with inappropriate antimicrobials. Therefore, risk factors for colonization or infection with multidrug-resistant strains of *Acinetobacter *spp. should be recognized, including ICU admission, previous colonization with methicillin-resistant *Staphylococcus aureus* (MRSA), beta-lactam, beta-lactam/beta-lactamase inhibitor and carbapenem antibiotics use, bedridden status, previous intensive care admission, central venous catheter, surgery, mechanical ventilation, hemodialysis, and malignancy [43–46]. For infections caused by multidrug-resistant *Acinetobacter* spp., antibiotic choices were usually limited. Options could be colistin [47–49] and tigecycline [50, 51]. Nevertheless, it showed in our study that colistin was only significantly associated with appropriate antimicrobial therapy. This was due to the fact that broad-spectrum antibacterial activity of colistin should be against most gram-negative bacteria whether they were pathogens of either community- or hospital-acquired infections. Other antimicrobials did not have adequate broad-spectrum antibacterial activity compared to colistin, especially when the MDR gram-negative bacteria were the causative agents. However, *Pseudomonas aeruginosa* did not beget inappropriate antimicrobial problems in our study.

 Drug resistant gram-positive bacteria, such as the most frequent MRSA, also significantly associated with the inappropriateness of empirical antimicrobials. Paul et al. demonstrated that inappropriate antimicrobials in MRSA septicemia obviously increased a risk for mortality [52]. Risk factors for infections with MRSA, including previous antibiotic use, prolonged hospitalization, intensive care, invasive devices, hemodialysis, MRSA colonization, and proximity to others with MRSA colonization or infection, and HIV infection should be concerned in order to select the better appropriate empirical antimicrobials [53–55]. The recommended initial antimicrobial management of MRSA bacteremia was intravenous vancomycin [56]. Nevertheless, our study showed no benefits of combination antimicrobial therapy on the appropriateness of antimicrobials and lower mortality, which contrasted to the studies by Kumar et al. [11, 57].

 Therefore, risk factors for drug-resistant organisms as well as local patterns of antimicrobial susceptibility should be of utmost concern in selecting the empiric regimen [29–31, 58–60]. Particularly, in the cases of critically ill patients, septic shock, high APACHE II score, leukopenia, respiratory or intra-abdominal infections, and nosocomial infection, physicians should be alert with particular attention on choosing appropriate initial empiric antimicrobials due to high mortality in these subgroups.

The education program and antimicrobial treatment guideline [61] should emphasize the knowledge of appropriate antimicrobial therapy administration. Otherwise, better empiric coverage of antimicrobial therapy could be obtained through immediate consultation with infectious disease specialists, such as “ID Fast Track” (like stroke or ST elevation myocardial infarction fast track) or antibiotic management teams [62] in some institutions [63–65]. As shown in our study, the adjustment of 24-hour antimicrobial therapy significantly resulted in a lower rate of inappropriateness than the first dose of antimicrobial therapy. Nevertheless, there were the controversies of the impact on early and appropriate antimicrobial therapy on lower mortality in some studies [66–68].

The delayed initiation of effective antimicrobial therapy following the onset of sepsis also increased mortality. The Surviving Sepsis Campaign's guideline recommended that the appropriate antimicrobial therapy should be administered within one hour of severe sepsis or septic shock recognition [5]. The recent study confirmed the significantly higher mortality associated with duration from triage or qualification for early goal-directed therapy more than one hour [3]. Our study showed the association between higher mortality and delay in giving antimicrobial therapy over one hour after the sepsis onset. However, there was statistical difference in mortality if the first-dose antimicrobial therapy was delayed more than three hours. To this reason, the appropriate antimicrobial therapy should be immediately initiated in patients with diagnosis of sepsis to achieve lower mortality.

For this purpose, the antimicrobial guideline for sepsis patients, development of strategies such as an immediately available antibiotics in the emergency department and critical wards, immediate “sepsis fast track consultants,” and antibiotic management teams should be accomplished to eliminate the delayed timing for antimicrobial medications and thus to ensure the timely administration of appropriate empiric antimicrobial therapy in sepsis patients in order to reduce the morbidity and mortality.

### 4.1. Limitation

The limitations of this study were retrospective methodology and the inclusion of patients only from medical wards of an academic referral center/tertiary medical center, which may not reflect the entire population in other departments or hospitals.

## 5. Conclusion

Inappropriate empirical antimicrobial therapy frequently occurs in about 29.7% of septic bacteremic patients, and it is associated with 75% mortality rate. Important risk factors of inappropriate antimicrobials including nosocomial infection, leukopenia, and drug-resistant organisms should be concerned to achieve better appropriateness. Furthermore, the mortality rate could be significantly increased by the delayed timing of first-dose antimicrobial therapy more than 3 hours after the diagnosis of sepsis. Thus, more efforts must be employed to increase the appropriateness of initial antimicrobial therapy and decrease timing from sepsis diagnosis to initiation of antimicrobials in a bid to reduce the mortality in patients with sepsis.

## Figures and Tables

**Table 1:** Baseline characteristics of septic patients with bacteremia by appropriateness of empiric antimicrobial therapy.

Baseline characteristics	All patients (*n* = 229)	Inappropriate first-dose antimicrobial therapy (*n* = 68)	Appropriate first-dose antimicrobial therapy (*n* = 161)
Male no. (%)	114 (49.8)	37 (54.4)	77 (47.8)
Age			
(i) Year—mean (SD.)	63.5 (17.2)	64.6 (18.4)	63.0 (16.8)
(ii) 65–79 yr.—no. (%)	65 (28.4)	21 (30.9)	44 (27.3)
(iii) ≥80 yr.—no. (%)	46 (20.1)	15 (22.1)	31 (19.3)

No. of comorbid illness—no. (%)			
(i) Presence of co-morbid illnesses	222 (96.9)	66 (97.1)	156 (96.9)
(ii) 1 illness	30 (13.1)	6 (8.8)	24 (14.9)
(iii) 2 illnesses	53 (23.1)	14 (20.6)	39 (24.2)
(iv) ≥3 illnesses	139 (60.7)	46 (67.6)	94 (57.8)

Type of co-morbid illness—no. (%)			
(i) Diabetes mellitus	71 (31.0)	18 (26.5)	53 (32.9)
(ii) Immunosuppressive^∗^	67 (29.3)	19 (27.9)	48 (29.8)
(iii) Reduced mobility	68 (29.7)	20 (29.4)	48 (29.8)
(iv) Liver failure^∗∗^	50 (21.8)	16 (23.5)	34 (21.1)
(v) Congestive heart failure NYFC 3-4 EF < 40%	50 (21.8)	14 (20.6)	36 (22.4)
(vi) Chronic kidney disease (serum creatinine ≥1.5 mg/dL)	42 (18.3)	16 (23.5)	26 (16.1)
(vii) Hematologic malignancies	42 (18.2)	9 (13.2)	33 (20.5)
(viii) Neutropenia (<500 cells/uL)	41 (17.9)	11 (26.8)	30 (18.6)
(ix) Metastatic solid cancer	33 (14.4)	10 (14.7)	23 (14.3)
(x) Chronic dialysis dependence (HD/PD)	16 (7.0)	5 (7.4)	11 (6.8)
(xi) COPD^∗∗∗^	14 (6.1)	4 (5.9)	10 (6.2)
(xii) AIDS (1993 CDC criteria)^∗∗∗∗^	11(4.8)	8 (11.8)	3 (1.9)
(xiii) Postoperative status	13 (5.7)	1 (1.5)	12 (7.5)

^∗^Immunosuppressive chemotherapy, radiation, or long-term steroid therapy ≥10 mg prednisone equivalent/day.

^∗∗^Biopsy-proven cirrhosis, documented variceal hemorrhage or portal hypertension, hepatic ascites, or encephalopathy.

^∗∗∗^Medication or oxygen requiring chronic restrictive, obstructive or vascular disease resulting in severe exercise restriction, that is, unable to climb stairs or perform household duties. Documented chronic hypoxia, hypercapnia, secondary polycythemia, severe pulmonary hypertension > 40 mmHg, respiratory dependency

^∗∗∗∗^ *P* = 0.001.

**Table 2:** Type and site of infection and severity of sepsis by appropriateness of empiric antimicrobial therapy.

Infection/sepsis	All patients (*n* = 229)	Inappropriate first dose of antimicrobial therapy (*n* = 68)	Appropriate first dose of antimicrobial therapy (*n* = 161)	Inappropriate 24-hour antimicrobial therapy (*n* = 58)	Appropriate 24-hour antimicrobial therapy (*n* = 171)
Type of infection—no. (%)					
(i) Community- acquired^∗^	63 (27.5)	10 (14.7)	53 (32.9)	9 (14.3)	54 (85.7)
(ii) Healthcare associated	85 (37.1)	29 (42.6)	56 (34.8)	19 (22.3)	66 (77.6)
(iii) Hospital-acquired	81 (35.4)	29 (42.6)	52 (32.3)	30 (37.0)^∗^	51 (63.0)

Site of infection—no. (%)					
(i) Respiratory	75 (32.8)	23 (33.8)	52 (32.3)		
(ii) Intra-abdominal	54 (23.6)	14 (20.6)	40 (24.8)		
(iii) Genitourinary^∗^	47 (20.5)	20 (27.9)	27 (16.8)		
(iv) Primary blood stream	19 (8.3)	7 (10.3)	12 (7.5)		
(v) Skin and soft tissue^∗^	18 (7.9)	17 (10.6)	1 (1.5)		
(vi) Intravascular catheter	9 (3.9)	1 (1.5)	8 (5.0)		
(vii) Surgical site	3 (1.3)	2 (2.9)	1 (0.6)		
(viii) Cardiovascular	2 (0.9)	0	2 (1.2)		
(ix) Bone and joint	2 (0.9)	0	2 (1.2)		

Severity of sepsis—no. (%)					
APACHE II score, unit mean (SD)	24.7 (6.8)	24.6 (7.0)	24.7 (6.8)		
APACHE II score 25–30 unit no. (%)	64 (27.9)	21 (30.9)	43 (26.7)		
APACHE II score >30 unit no. (%)	48 (21.0)	14 (20.6)	34 (21.1)		
(i) Sepsis	31 (13.5)	13 (19.1)	18 (11.2)		
(ii) Severe sepsis	58 (25.3)	16 (23.5)	42 (26.1)		
(iii) Septic shock^∗^	140 (61.1)	39 (57.4)	101 (62.7)		

*P < 0.05.

**Table 3:** Type of microorganism isolated from blood by appropriateness of empiric antimicrobial therapy.

Type of bacteria	All patients (*n* = 229)	Inappropriate first dose of antimicrobial therapy (*n* = 68)	Appropriate first dose of antimicrobial therapy (*n* = 161)
Gram-positive bacteria—no. (%)	63 (27.5)	13 (19.1)	50 (31.1)
*Streptococcus pneumonia*	2 (0.9)	0	2 (1.2)
*Streptococcus* gr. B, C, D, G	12 (5.2)	0	12 (7.5)
Alpha-hemolytic *Streptococcus *	7 (3.1)	0	7 (4.3)
MSSA^∗^	20 (8.7)	1 (1.5)	19 (11.8)
MRSA^∗^	11 (4.8)	7 (10.3)	4 (2.5)
CNSA	3 (1.3)	1 (1.5)	2 (1.2)
*Enterococcus faecalis*	1 (0.4)	0	1 (0.6)
*Enterococcus faecium* ^ ∗^	5 (2.2)	4 (5.9)	1 (0.6)

Gram-negative bacteria^∗^—no. (%)	166 (72.5)	55 (80.9)	111 (68.9)
*Acinetobacter* spp.^∗∗^	28 (12.2)	17 (25.0)	11 (6.8)
*Escherichia coli*	66 (28.8)	25 (38.8)	41 (25.5)
(i) ESBL-positive^∗∗^	28 (12.2)	24 (35.3)	4 (2.5)
*Klebsiella pneumoniae* ^ ∗^	29 (12.7)	4 (5.9)	25 (15.5)
(i) ESBL-positive	7 (3.1)	4 (5.9)	3 (1.9)
*Proteus mirabilis*	5 (2.2)	1 (1.5)	4 (2.5)
(i) ESBL-positive	1 (0.4)	0	1 (0.6)
*Pseudomonas aeruginosa*	10 (4.4)	1 (1.5)	9 (5.6)
*Salmonella* spp.	10 (4.4)	3 (4.4)	7 (4.3)
Other non-fermentative gram-negative bacilli	9 (3.9)	3 (4.4)	6 (3.7)
*Enterobacter* spp.	3 (1.3)	1 (1.5)	2 (1.2)
Other gram-negative bacteria^∗∗∗^	8 (3.5)	0	8 (5.0)

^∗^ *P* < 0.05.

**P < 0.001.

^∗∗∗^Organism (*N*); *Vibrio* spp. (2), *Aeromonas* spp. (2), *Stenotrophomonas maltophilia* (1)*, Burkholderia pseudomallei* (1), *Citrobacter koseri* (1), *Roseomonas* spp. (1).

**Table 4:** Type of first-dose empiric antimicrobials for sepsis by appropriateness of empiric antimicrobial therapy.

Type of antimicrobials (first dose)	All patients (*n* = 229)	Inappropriate first dose of antimicrobial therapy (*n* = 68)	Appropriate first dose of antimicrobial therapy (*n* = 161)
Mono- or combination therapy			
Monotherapy	145 (63.3)	45 (66.2)	100 (62.1)
Combination	82 (35.8)	21 (30.9)	61 (37.9)
3rd generation cephalosporin	100 (43.7)	29 (42.6)	71 (44.1)
4th generation cephalosporin	32 (14.0)	9 (13.2)	23 (14.3)
Carbapenems	53 (23.1)	17 (25.0)	36 (22.4)
(i) Imipenem	26 (11.4)	7 (10.3)	19 (11.8)
(ii) Meropenem	24 (10.5)	10 (14.7)	14 (8.7)
(iii) Doripenem	1 (0.4)	0	1 (0.6)
Beta-lactam/beta-lactamase inhibitor^∗^	28 (12.2)	6 (8.8)	22 (13.7)
Vancomycin	26 (11.4)	10 (14.7)	16 (9.9)
Aminoglycosides	17 (7.4)	5 (7.4)	12 (7.5)
Fluoroquinolones	19 (8.3)	7 (10.3)	12 (7.5)
(i) Ciprofloxacin	13 (5.7)	4 (5.9)	9 (5.6)
(ii) Levofloxacin	6 (2.6)	3 (4.4)	3 (1.9)
Colistin^∗∗∗^	11 (4.8)	0	11 (6.8)

*Piperacillin/tazobactam.

**Amikacin.

***P < 0.05.

**Table 5:** Univariate analysis of factors associated with 28-day and overall mortality.

Factors	Total *n* = 229*n* (%)	28-day mortality *n* = 143*n* (%)	*P* value	OR (95% CI)	Overall mortality *n* = 161*n* (%)	*P* value	OR (95% CI)
Age > 65 yrs	111 (48.5)	74 (66.7)	0.221	1.42 (0.83–2.43)	86 (77.5)	**0.030**	1.97 (1.10–3.53)
Nosocomial infection	81 (35.4)	55 (67.9)	0.108	1.44 (0.82–2.55)	65 (80.2)	**0.016**	2.20 (1.16–4.18)
Septic shock	140 (61.1)	96 (68.6)	**0.017**	1.95 (1.13–3.38	102 (72.9)	0.200	1.36 (0.77–2.43)
Inappropriate first dose	68 (42.79)	46 (67.6)	0.292	1.38 (0.76–2.51)	51 (75.0)	0.213	1.39 (0.73–2.64)
Inappropriate 24-hour dose	58 (25.33)	38 (65.5)	0.176	1.19 (0.64–2.23)	43 (74.1)	0.160	1.29 (0.66–2.52)
Duration from sepsis until first-dose antimicrobial therapy > 3 hours	117 (51.1)	79 (67.5)	0.106	1.56 (0.91–2.67)	90 (76.9)	**0.026**	1.93 (1.08–3.43)
Wbc ≤ 4,000 per uL	40 (17.5)	33 (82.5)	**0.004**	3.39 (1.43–8.04)	34 (85.0)	**0.035**	2.77 (1.10–6.94)
Plt ≤ 100,000 per uL	109 (47.6)	82 (75.2)	**<0.001**	2.94 (1.67–5.16)	88 (80.7)	**0.001**	2.698 (1.48–4.92)
HCO_3_^−^ ≤ 20 mEq/L	135 (59.0)	92 (68.1)	**0.038**	1.80 (1.05–3.11)	96 (71.1)	0.770	1.10 (0.62–1.95)
Albumin ≤ 3.2 g/dL	171 (74.7)	117 (68.4)	**0.002**	2.67 (1.45–4.91)	128 (74.9)	**0.013**	2.26 (1.21–4.21)
APACHE II score ≥ 25	100 (43.7)	79 (79.0)	**<0.001**	3.82 (2.11–6.90)	81 (81.0)	**0.002**	2.61 (1.41–4.82)
Serum cortisol ≥ 35 mg/dL (*n* = 64)	38 (59.4)	29 (76.3)	**0.009**	4.39 (1.49–12.93)	30 (78.9)	**0.029**	3.75 (1.25–11.21)
Serum lactate ≥ 2.2 mmol/L (*n* = 31)	26 (83.9)	20 (76.9)	**0.027**	13.33 (1.24–143.15)	20 (76.9)	**0.027**	13.33 (1.24–143.15)
Comorbidity > 4	84 (36.7)	61 (42.7)	**0.017**	2.04 (1.14–3.64)	69 (42.9)	**0.003**	2.65 (1.38–5.09)
Liver failure	50 (21.8)	38 (26.6)	**0.031**	2.23 (1.09–4.56)	42 (26.1)	**0.022**	2.65 (1.17–5.99)
Congestive heart failure	50 (21.8)	38 (26.6)	**0.031**	2.23 (1.09–4.56)	41 (25.5)	0.053	2.24 (1.02–4.92)
High-risk source^∗^	129 (56.3)	89 (69.0)	**0.027**	1.90 (1.1–3.26)	101 (78.3)	**0.003**	2.41 (1.35–4.29)
Respiratory	75 (32.8)	54 (72.0)	**0.042**	1.88 (1.03–3.41)	64 (85.3)	**<0.001**	3.42 (1.67–7.01)
Intra-abdomen	54 (23.6)	35 (64.8)	0.749	1.14 (0.61–2.16)	37 (68.5)	0.736	0.90 (0.46–1.73)
Genitourinary	47 (20.5)	20 (14.0)	**0.002**	0.36 (0.18–0.69)	24 (51.0)	**0.002**	0.34 (0.18–0.67)
Skin and soft tissue	18 (7.9)	11 (61.1)	1.000	0.94 (0.35–2.53)	12 (66.7)	0.789	0.83 (0.30–2.32)
Monotherapy-inappropriate	45 (19.65)	28 (62.2)	0.632	1.19 (0.58–2.45)	32 (71.1)	0.623	1.21 (0.56–2.61)
Monotherapy-appropriate	100 (43.67)	58 (58.0)			67 (67.0)		
Combination-inappropriate	21 (9.17)	16 (76.2)	0.306	1.81 (0.58–5.60)	17 (81.0)	0.355	1.78 (0.53–6.03)
Combination-appropriate	61 (26.64)	39 (63.9)			43 (70.5)		

^∗^Respiratory and Intra-abdominal [15, 16].

**Table 6:** Multivariate analysis of factors associated with inappropriate first-dose and 24-hour antimicrobial therapy.

Factors	Total *n* (%)	First dose	OR (95% CI)	24-hour dose	OR (95% CI)
		*P* value		*P* value	
Nosocomial infection	81 (35.4)	0.225	1.86 (0.68–5.04)	**0.018 **	3.31 (1.22–8.93)
Neutropenia	41 (17.9)	**0.022 **	2.94 (0.16–7.14)	0.156	0.51 (0.20–1.29)
WBC < 4000 per uL	40 (17.5)	**0.016 **	2.45 (1.18–5.08)	0.121	1.81 (0.86–3.84)
Platelet < 100,000 per uL	109 (47.6)	**0.013 **	2.42 (1.20–4.88)	0.055	2.13 (0.98–4.62)
Drug-resistant gram-positive bacteria	17 (7.4)	**<0.001 **	6.03 (2.35–15.43)	**<0.001 **	6.14 (2.30–16.37)
Drug-resistant gram-negative bacteria	74 (32.3)	**<0.001 **	10.76 (3.98–29.09)	**<0.001 **	8.50 (3.10–23.32)

Other variables: Age ≥ 65 years, neutropenia, chronic renal failure, albumin ≤ 3.2 g/dL, serum cortisol ≥ 35 mg/dL, co-morbidity ≥ 4, APACHE II score ≥ 25, septic shock, congestive heart failure, liver failure, respiratory infection, genitourinary infection, antimicrobial mono-therapy, and colistin usage.

**Table 7:** Multivariate analysis of factors associated with 28-day and overall mortality.

Factors	Total *n* = 229 *N* (%)	Overall mortality (*P* value)	OR (95% CI)	28-day mortality (*P* value)	OR (95% CI)
Inappropriate first dose	68 (29.7)	**0.049 **	2.52 (1.01–6.32)	0.059	2.48 (0.97–6.36)
Inappropriate 24-hour dose	58 (25.3)	0.251	0.61 (0.26–1.42)	0.323	0.65 (0.28–1.53)
Duration from sepsis until first-dose antimicrobial therapy >3 hours	117 (51.1)	**0.009 **	1.80 (1.16–2.80)	**0.006 **	1.94 (1.21–3.10)
Congestive heart failure	50 (21.8)	**<0.001 **	2.07 (1.39–3.10)	**<0.001 **	2.17 (1.43–3.30)
Age > 65 years	111 (48.5)	**0.022 **	1.55 (1.07–2.26)	**0.114 **	1.38 (0.93–2.05)
Platelet < 100,000 per uL	109 (47.6)	**0.010 **	1.67 (1.13–2.46)	**0.029 **	1.55 (1.05–2.30)
Serum albumin < 3.2 g/dL	171 (74.7)	**0.016 **	1.74 (1.11–2.74)	**0.030 **	1.71 (1.05–2.76)
APACHE II score ≥ 25	100 (43.7)	**0.011 **	1.66 (1.13–2.44)	**<0.001 **	2.12 (1.41–3.19)
Sepsis shock	140 (61.1)	**0.001 **	1.93 (1.30–2.87)	**0.009 **	1.75 (1.15–2.65)

Other variables: infection acquisition site (community or nosocomial); source of infection; septic shock; APACHE II scores; major co-morbidities (neutropenia and liver failure); predictive of mortality laboratories (white blood cell less than 4000 per mm^3^); drug-resistant organism.
